# Global pattern of phytoplankton diversity driven by temperature and environmental variability

**DOI:** 10.1126/sciadv.aau6253

**Published:** 2019-05-15

**Authors:** Damiano Righetti, Meike Vogt, Nicolas Gruber, Achilleas Psomas, Niklaus E. Zimmermann

**Affiliations:** 1 Environmental Physics, Institute of Biogeochemistry and Pollutant Dynamics, ETH Zurich, 8092 Zurich, Switzerland.; 2 Dynamic Macroecology, Landscape Dynamics, Swiss Federal Research Institute WSL, 8903 Birmensdorf, Switzerland.; 3 Department of Environmental Systems Science, ETH Zurich, 8092 Zurich, Switzerland.

## Abstract

Despite their importance to ocean productivity, global patterns of marine phytoplankton diversity remain poorly characterized. Although temperature is considered a key driver of general marine biodiversity, its specific role in phytoplankton diversity has remained unclear. We determined monthly phytoplankton species richness by using niche modeling and >540,000 global phytoplankton observations to predict biogeographic patterns of 536 phytoplankton species. Consistent with metabolic theory, phytoplankton richness in the tropics is about three times that in higher latitudes, with temperature being the most important driver. However, below 19°C, richness is lower than expected, with ~8°– 14°C waters (~35° to 60° latitude) showing the greatest divergence from theoretical predictions. Regions of reduced richness are characterized by maximal species turnover and environmental variability, suggesting that the latter reduces species richness directly, or through enhancing competitive exclusion. The nonmonotonic relationship between phytoplankton richness and temperature suggests unanticipated complexity in responses of marine biodiversity to ocean warming.

## Teaser

In situ data and modeling offer a first global view of the spatial pattern of phytoplankton species richness in the ocean.

## INTRODUCTION

Marine phytoplankton dominate primary production across ~70% of Earth’s surface (*1*), play a pivotal role in channeling energy and matter up the food chain, and control ocean carbon sequestration (*2*). The diversity of phytoplankton species in open waters has intrigued ecologists for at least half a century (*3*), but the global pattern of this diversity and its underlying drivers have been unclear (*4*, *5*). This is a critical gap in our understanding of the oceans since the richness of phytoplankton species, a key element of their diversity, may enhance resource use efficiency (*6*), and thus primary production, as often seen in terrestrial systems (*7*). For those oceanic taxa that have been investigated, including foraminifera, fish, and invertebrates, species richness tends to peak at low to mid-latitudes and to decline sharply toward the poles (*8*–*11*). This decline is consistent with the metabolic theory of ecology (*12*, *13*); i.e., the hypothesis that temperature exerts a key control on metabolic rates and thus promotes speciation and increased species richness in warm tropical areas through time (*14*). However, a recent large-scale study on marine phytoplankton richness is at odds with the prediction by metabolic theory (*4*), and latitudinal richness gradients identified for individual phytoplankton groups have taken various shapes (*5*, *15*–*17*). Observed discrepancies may originate from other factors such as resource competition (*17*, *18*), differences in body size (*15*, *19*), or undersampling of richness (*20*). Empirical tests of the competing theories explaining global diversity patterns have so far been impeded by the paucity of in situ observations and the lack of systematic sampling schemes for open-ocean phytoplankton (*21*).

Here, we overcome these limitations and provide the first analysis of marine phytoplankton species richness and its ecological drivers at the global scale, using 1,056,363 presence observations of 1300 species compiled from multiple sources as the basis for our analysis. These data span all major taxa, ocean basins, latitudes, and most seasons (fig. S1). To address the strong spatial and seasonal bias in sampling effort, we analyze the subset of data of species with at least 24 presences (553 species and 699,387 observations) using species distribution models (SDMs), which have been set up specifically to account for uneven sampling and fit each species’ ecological niche as a function of multiple environmental predictors. Our statistical approach thus aims at filtering out spurious patterns in the raw data while integrating all observational evidence. We successfully build probabilistic SDMs (fig. S2) for 536 species using generalized additive models (GAM) and use generalized linear models (GLM) and random forest models (RF) to assess the robustness of our findings. We project the species’ niches to the global ocean at a 1° resolution and at monthly scales and diagnose richness from the overlap of species’ presence-absence projections. Since the diversity of short-lived phytoplankton may change over the course of each year, we project species richness for each month and map its annual mean state, as well as monthly species turnover (see Materials and Methods).

## RESULTS AND DISCUSSION

**Fig. 1:**
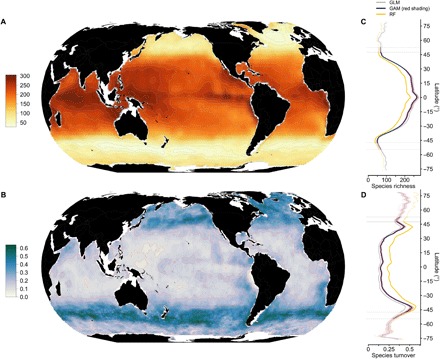
Global patterns of monthly phytoplankton species richness and species turnover. (**A**) Annual mean of monthly species richness and (**B**) month-to-month species turnover projected by SDMs. Latitudinal gradients of (**C**) richness and (**D**) turnover. Colored lines (regressions with local polynomial fitting) indicate the means per degree latitude from three different SDM algorithms used (red shading denotes ±1 SD from 1000 Monte Carlo runs that used varying predictors for GAM). Poleward of the thin horizontal lines shown in (C) and (D), the model results cover only <12 or <9 months, respectively.

**Fig. 2:**
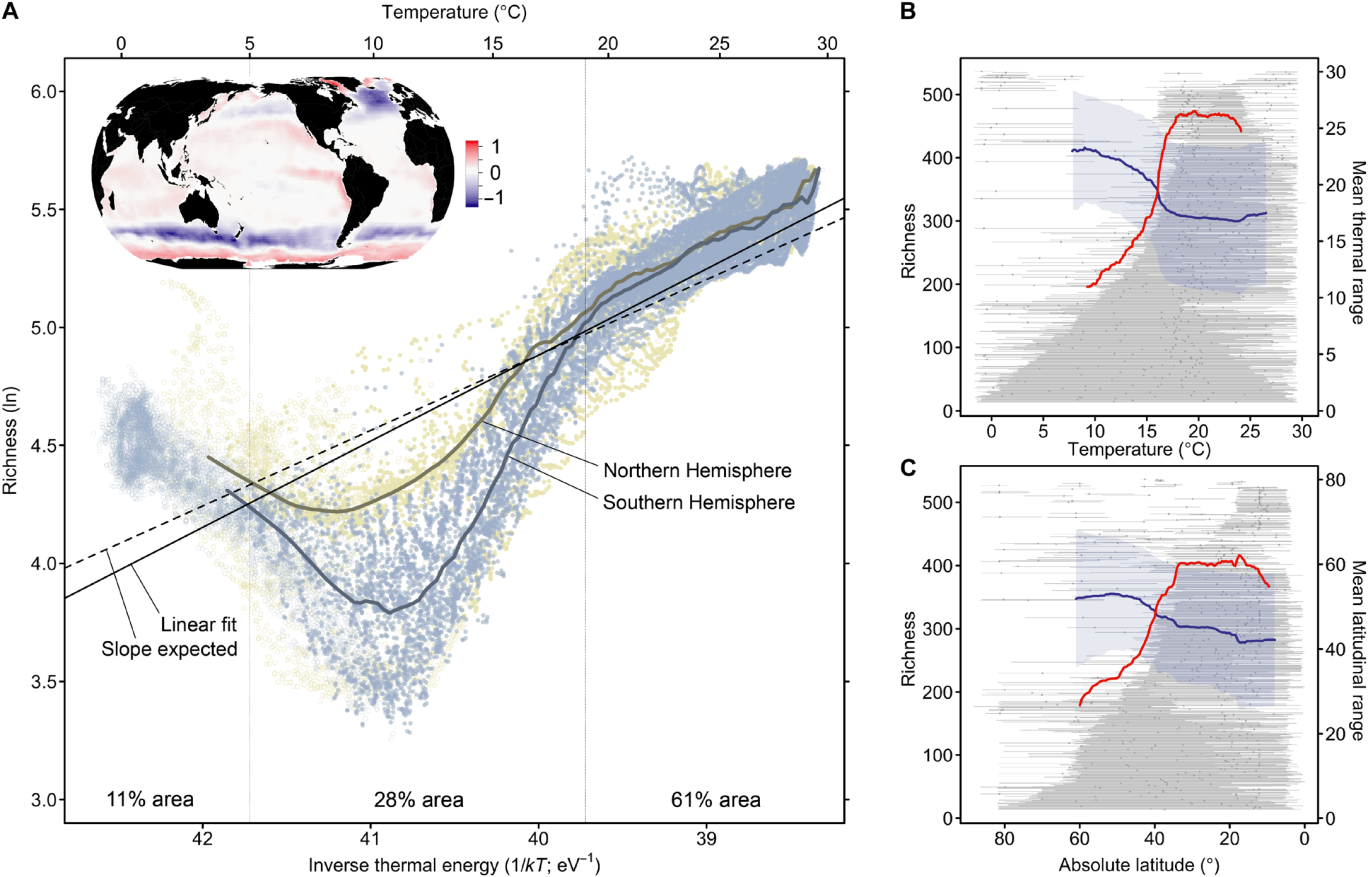
Relationships between species richness and temperature or latitude. (**A**) The natural logarithm of the annual mean of monthly phytoplankton richness is shown as a function of sea temperature (*k*, Boltzmann’s constant; *T*, temperature in kelvin). Filled and open circles indicate areas where the model results cover 12 or less than 12 months, respectively. Trend lines are shown separately for each hemisphere (regressions with local polynomial fitting). The solid black line represents the linear fit to richness, and the dashed black line indicates the slope expected from metabolic theory (−0.32). The map inset visualizes richness deviations from the linear fit. The relative area of three different thermal regimes (separated by thin vertical lines) is given at the bottom of the figure. Observed thermal (**B**) and latitudinal (**C**) ranges of individual species are displayed by gray horizontal bars (minimum to maximum, dots for median) and ordered from wide-ranging (bottom) to narrow-ranging (top). The *x* axis in (C) is reversed for comparison with (B). Red lines show the expected richness based on the overlapping ranges, and blue lines depict the species’ average range size (±1 SD, blue shading) at any particular *x* value. Lines are shown for areas with higher confidence.

**Fig. 3:**
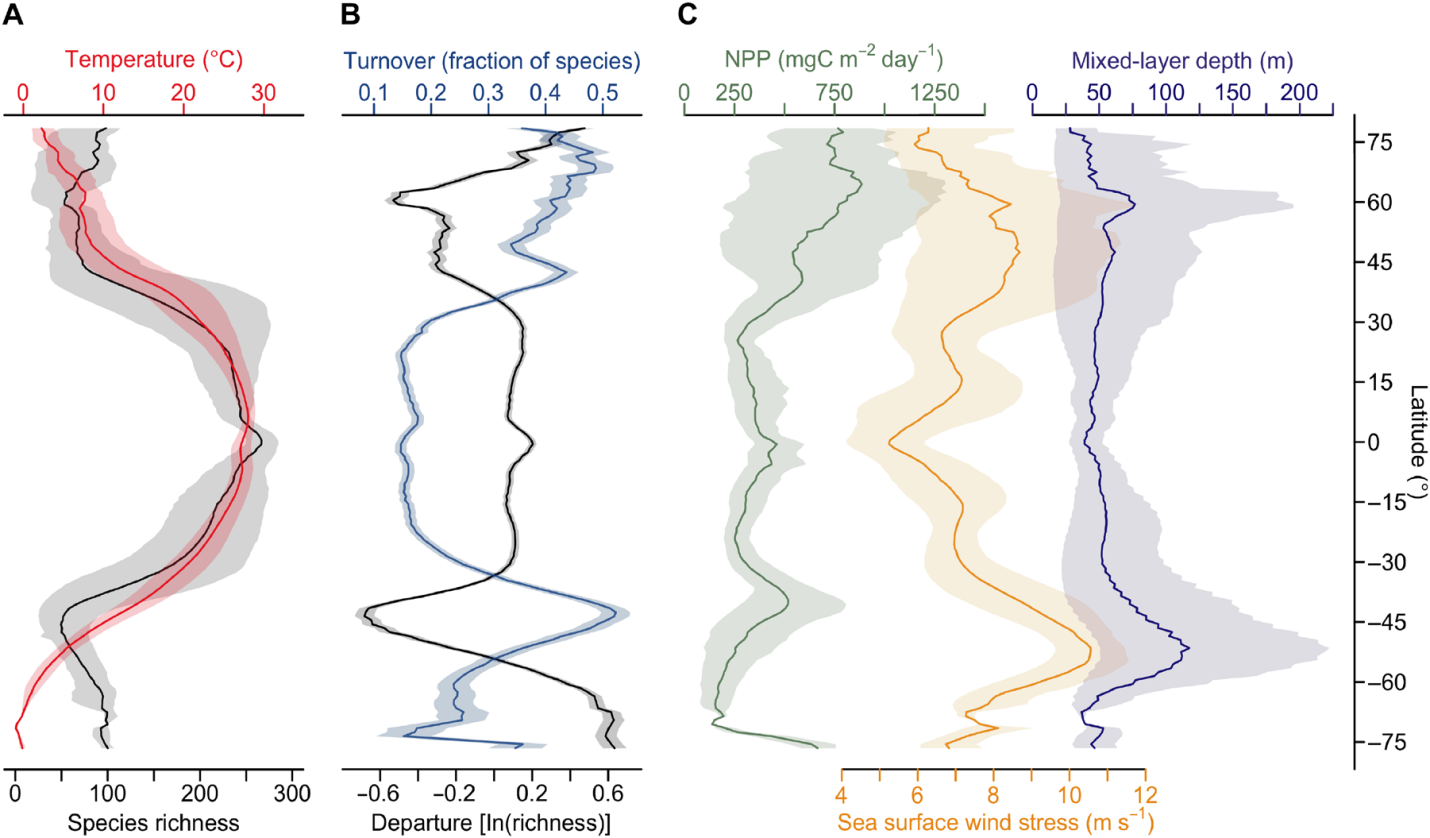
Latitudinal trends in phytoplankton richness and selected environmental variables. (**A**) Annual mean of monthly species richness (black line) and sea temperature (red line). Shadings indicate the annual amplitude of monthly richness (gray) and temperature (red). (**B**) Departure of richness (black line) from the linear fit (see Fig. 2A) versus species turnover (blue line) by latitude. Shadings denote ±1 SD from Monte Carlo runs. (**C**) Net primary production (NPP; green), sea surface wind stress (orange), and mixed-layer depth (slate blue). Shadings indicate the annual amplitude (minimum to maximum of monthly patterns) for each variable.

Our diagnosed annual mean of monthly phytoplankton richness varies strongly with latitude, while longitudinal differences are comparably small (Fig. 1A). Phytoplankton richness is highest and least variable throughout the year in the inner tropics (<5°N and S; Fig. 3A), reaching more than 240 species on average. Richness hotspots, where roughly half of the total species analyzed occur simultaneously, are found in the central Indian, the equatorial and west Pacific, and the Indo-Australian Archipelago (Fig. 1A). Thus, hotspots of phytoplankton richness tend to be more tropical than those of foraminifera and other oceanic taxa (*8*, *9*). Analyzed by latitude, richness declines steeply poleward of 30° (Fig. 1C), reaches its minima (~50 species) and associated inflexion points at mid-latitudes (~45° to 65°N and ~45°S), and increases slightly toward the poles. This latitudinal pattern is composed of species with notable wide thermal ranges (15.8° ± 6.8°C, mean ± SD; Fig. 2B) and broad geographic distributions (Fig. 2C), with more than 60% of high-latitude species (>70°N and S) recorded close to the equator as well (table S1). The latitudinal ranges of most species (*n* > 400) tend to be aggregated between 30°N and 30°S, with relatively few specialist species populating the extratropics (Fig. 2C).

The raw observations corroborate the global latitudinal richness gradient at monthly resolution (fig. S4). The global richness pattern (Fig. 1A), which is diagnosed from these observations by our models, shows seasonal variation that is strongest from subtropical to temperate latitudes (Fig. 3A). Yet, its general form and, in particular, inflexion points (Fig. 1C) are persistent throughout the year and robust to corrections applied to balance the representation of major taxa in our model analysis (i.e., the proportion of species of a specific taxon captured in our SDMs; fig. S3A). Moreover, the diagnosed richness pattern is decoupled from the spatial density of phytoplankton raw observations (*R*^2^ = 0.015, *P* < 0.001, *n* = 536 species), robust against the model algorithm used (GAM versus GLM, *R*^2^ = 0.99, *P* < 0.001; GAM versus RF, *R*^2^ = 0.99, *P* < 0.001), and largely shared between major taxa (diatom versus dinoflagellate richness, *R*^2^ = 0.75, *P* < 0.001; diatom versus haptophyte richness, *R*^2^ = 0.53, *P* < 0.001; haptophyte versus dinoflagellate richness, *R*^2^ = 0.83, *P* < 0.001). Last, the diagnosed latitudinal richness gradient does not depend on the particular predictor set used in SDMs (Fig. 1C; red shading) and is also robust to choices with respect to the sampling of environmental data for SDMs, here referred to as environmental background (fig. S3B). While we consider the overall gradient as robust, the slopes poleward of ~50° latitude are prone to larger uncertainty, as winter months lack predictor data in these areas (Fig. 1C).

Sea surface temperature is the most important driver for phytoplankton richness in our data. It explains more than two-thirds of the global variation in diagnosed richness (*R*^2^ = 0.76, *P* < 0.001) and is the most powerful predictor for species richness in the underlying raw observations (table S2). Temperature is also the single most important environmental predictor for habitat suitability at the species level (table S2).

The strong role of temperature is consistent with the metabolic theory of ecology (*12*), but while this theory implies a single negative and linear relationship with a slope of −0.32 between logarithmic species richness and inverse thermal energy (*22*, *23*), we find three distinctly different regimes across the global ocean (Fig. 2A). Results match metabolic theory best for the ~60% of the ocean surface with annual mean temperatures above 19°C, with a linear regression slope of −0.37 (*R*^2^ = 0.65, *P* < 0.001). Between ~19° and 11°C, the slope steepens to −1.14 (*R*^2^ = 0.69, *P* < 0.001), and it reverses below ~11°C, with a value of 0.48 (*R*^2^ = 0.56, *P* < 0.001). Thus, monthly phytoplankton richness forms a V-shaped pattern at intermediate to low temperatures. This nonmonotonic relationship is similarly evident in the raw observations (fig. S5, A to E) and consistent with results based on independent in situ data (fig. S5F).

The globally nonmonotonic relationship between phytoplankton richness and temperature (Fig. 2A) suggests that temperature and the metabolic theory of ecology (*12*) provide incomplete explanations for the global-scale variation in phytoplankton richness. We next test whether such variation can be explained by the “physiological tolerance hypothesis” (*24*), which posits that variation in richness is a function of the number of overlapping ecological niches of species. If species had strong affinities for either warm or cold, rather than intermediate temperatures, then a nonmonotonic richness response to temperature may emerge because fewer species tolerate mid-temperatures. On the basis of direct analysis of the thermal ranges of all species in the raw observations, we reject this hypothesis, as the richness obtained by overlapping and summing up these ranges declines monotonically below ~19°C (Fig. 2B). Alternatively, changes in the magnitude of the richness-temperature slope may be in line with metabolic theory but reflect distinct species pools with differing activation energies (*12*, *13*). For example, linear slopes shallower than those predicted by metabolic theory have been reported for both marine phytoplankton (*4*) and zooplankton (*10*) richness. We reject this second alternative because the sorting of the thermal ranges within global temperatures (Fig. 2B) does not indicate a thermal separation of species pools, and the ranges of numerous tropical species reach latitudes with temperatures below 19°C (Fig. 2C), suggesting rather smooth shifts in species composition. Thus, we interpret the low richness between ~8° and 14°C (Fig. 2A) as an intermittent, non–temperature-driven suppression of richness, relative to the linear slope predicted by metabolic theory (*12*). This interpretation is favored by the fact that the global linear fit to the modeled richness (slope, −0.37, *R*^2^ = 0.66, *P* < 0.001; Fig. 2A) and linear fits to richness in the underlying raw data (fig. S5, A to C) closely match the slope of −0.32 predicted by metabolic theory (*22*, *23*). The reversed richness slope at lowest temperatures may signal a relaxation of intensity in the factors driving this suppression.

What could lead to suppressed phytoplankton species richness at temperate latitudes? The most notable feature associated with the global pattern of phytoplankton species richness is its strong and negative correlation with month-to-month species turnover (*R*^2^ = 0.62, *P* < 0.001; Fig. 1B). This turnover quantifies the monthly change in species composition in each 1° cell, as projected by our SDMs. While turnover is lowest in the tropics with only ~15% in species being replaced on average between months, it strongly increases poleward of 20° and it peaks at ~40° latitude with monthly turnover rates approaching 50% (Fig. 1D). These areas of maximum turnover are congruent with areas characterized by low richness [Figs. 2A (map) and 3B]. In our analysis, maxima in turnover denote particularly strong month-to-month variability of those environmental factors that represent statistically determined key dimensions of species’ ecological niches. This suggests that temporal variability of the environment is a critical determinant of richness.

Seasonal and short-term environmental variability may reduce richness through abiotic factors, including strong turbulence in the water column, which may select for few species (*25*). This is supported by the negative relationship between richness and sea surface wind stress (*R*^2^ = 0.63, *P* < 0.001) or mixed-layer depth (MLD; *R*^2^ = 0.35, *P* < 0.001), two proxies for turbulence and light limitation in the water column with strong seasonality at mid-latitudes (Fig. 3C). Furthermore, we find progressively wider tolerance ranges of species in the observational data to increasing wind stress, deeper mixing, and higher nitrate levels (fig. S6, A, B, and F), suggesting a preferential selection for generalist species with broad niches in regions of strong environmental variability and elevated nutrient supply.

Competitive exclusion among species mediated by high temporal environmental variability (*26*) is an additional mechanism that may reduce richness at temperate latitudes. Strong seasonality in upper ocean stratification and associated variations in nutrients and light induce strong seasonality in productivity at mid- to high latitudes (Fig. 3C), with phytoplankton blooms dominated by relatively few species that monopolize local resources (*18*, *27*, *28*). While our presence-only raw data cannot reveal the dominance hierarchy of species in terms of abundance, our analysis suggests that low monthly richness at mid-latitudes is linked to rapid species turnover. These results provide the first confirmatory evidence to global mechanistic model results that proposed a negative influence of high temporal variability of the environment on phytoplankton richness, mediated via competitive exclusion (*26*) and transient blooms of opportunists (*29*). However, unlike our results, mechanistic simulations proposed lowest phytoplankton richness at highest (*26*) or subtropical (*29*) latitudes, with the exact location of diversity minima depending on the parameterization of trophic interactions.

Our modeling approach cannot disentangle the relative importance of different mechanisms in generating the latitudinal richness gradient identified. However, our results suggest that environmental variability strongly modifies global gradients in phytoplankton diversity (Fig. 1, C and D), in line with recent findings related to marine bacteria (*30*). Rapoport’s rule predicts an increase of species range size with increasing latitude (*31*) and has been used to explain a monotonically decreasing species richness from the equator to poles by an increase in seasonal environmental variability, selecting for wide-ranging species. We confirm a weak trend of increasing range size with increasing latitude (Fig. 2C) and a negative relationship between intra-annual variability and richness. Yet, the variability relevant for short-lived phytoplankton shows distinct mid-latitude peaks (Figs. 1D and 3C), which may explain the emergence of a latitudinal richness gradient that does not conform with the pattern expected by Rapoport’s rule.

We do not exclude the possibility that factors besides temperature (table S2) and environmental variability affect richness. Spatial trends in body size and abundance may lead to departures of richness from the slope predicted by metabolic theory (*12*) as recently discussed for a similar nonlinear pattern in freshwater phytoplankton (*32*). Furthermore, richness responses to temperature may level off in the nutrient-poor tropical sea due to a slowdown of metabolic rates under nutrient scarcity, despite high temperatures (*33*). However, tropical richness gradients emerge in our analysis in the absence of clear nutrient gradients (*34*), and continuously increasing nutrient concentrations below ~17°C (*34*) cannot explain the marked change in the richness trend at ~11°C (Fig. 2A). While the effect of environmental variability thus remains a valid hypothesis for this change, finer-scale data are needed to assess the relative roles of nutrients, variability, and temperature in structuring global phytoplankton richness.

In conclusion, synthesizing results across key taxa reveals a first global pattern of phytoplankton species richness in line with a strong role of temperature on the evolutionary outcome of species’ habitats and large-scale biodiversity (*12*, *13*, *22*). In addition, we demonstrate the critical role of environmental variability (*26*, *27*) for species turnover and diversity through time. Together, these mechanisms shape a nonmonotonic latitudinal gradient in monthly phytoplankton species richness that is distinctly different from its terrestrial autotrophic counterpart (*35*) and also from most other marine taxa (*8*–*11*). Climate change is expected to modify both the variability and annual averages in environmental conditions, including temperature and ocean stratification. Our study proposes a link of phytoplankton richness with both temperature and ocean variability; therefore, responses of global patterns in marine phytoplankton diversity to climate change may be more complex than hitherto anticipated, with possible impacts on higher trophic organisms, productivity, and ecosystem function.

## MATERIALS AND METHODS

To explore how phytoplankton diversity varies along environmental and spatial gradients, we compiled global phytoplankton presence data and oceanographic predictors. Phytoplankton presence data were used to calibrate SDMs, designed to compensate for data bias and sample scarceness. Direct analyses of these raw data served as a robustness test for the model results. Furthermore, we used independent phytoplankton data for validation of our results. All analyses were conducted using R language.

### Phytoplankton field data

We compiled phytoplankton data from the Global Biodiversity Information Facility (GBIF; https://www.gbif.org, retrieved on 7 December 2015), the Ocean Biogeographic Information System (OBIS; https://www.obis.org, retrieved on 5 December 2015), Villar *et al*. (*36*), and the MAREDAT initiative (table S3) (*37*). The final dataset contained 1,056,363 presence observations from 1298 species and two genera (collectively termed “species”), which were recorded at an average depth of 5.41 ± 6.95 m (mean ± SD) at 182,392 locations in space and time. Observation densities were spatially biased, with 49% of total observations originating from the north Atlantic and only 0.9% originating from the south Atlantic. Methods involved in original data collection included filters (*38*), microscopy (*36*, *39*), and flow cytometers (*37*), among others. We retrieved all species observations for seven phyla: *Cyanobacteria*, *Chlorophyta* (excluding macroalgae), *Cryptophyta*, *Myzozoa*, *Haptophyta*, *Ochrophyta*, and *Euglenozoa.* More specifically, among the *Ochrophyta*, we included the classes *Bacillariophyceae*, *Chrysophyceae*, *Pelagophyceae*, and *Raphidophyceae.* Among the *Myzozoa*, we considered the class *Dinophyceae*. Among the *Euglenozoa*, we considered the class *Euglenoidea*. In addition, we compiled observations of *Prochlorococcus* and *Synechococcus* from the data sources. These genera are globally abundant but rarely identified to a species level. We excluded records (i) if they were listed as “fossil specimen” or “preserved specimen,” (ii) if they were associated with year of collection >2015 or <1800, (iii) if they were associated with negative depths, or (iv) if they were associated with nonsensible coordinates. We removed data below the monthly climatological mixed layer based on the temperature criterion (*40*), as data at depth were insufficient to develop species models. However, for “mixed-layer species” (i.e., species recorded in the mixed layer), we assumed that data without depth indication stem from the mixed layer as well. Species names in the original data were harmonized following expert opinion (see Supplementary Materials and Methods). The dataset spanned all major phytoplankton taxa of the marine realm (*41*) and reflected, within the bounds of uncertainty, roughly similar factions of the total species known among key taxa (table S4).

Independent data (<2.9% of the observations overlap with the above dataset) (*39*), primarily based on Atlantic transect cruises, served for validation of results obtained from the main dataset. These independent data were collected on the basis of a consistent methodology by the same taxonomist, containing 4217 presence observations from 303 phytoplankton species within the mixed layer (*39*).

### Open-ocean definition

To reduce confounding influences of more complex and fertile coastal environments on our open ocean phytoplankton analyses, we excluded data from seas shallower than 200 m (*42*) and from seas with surface salinities below 20 (*43*).

### Environmental data

We compiled data on 10 environmental variables that represent key dimensions of phytoplankton ecological niches (*44*–*46*), which shape species’ distributions via effects on physiology, growth, and species competition (*17*, *25*, *47*). These variables served as candidate predictors for SDMs and as single predictors in species richness models. Variables were aggregated at a monthly (*n* = 12) climatological and globally gridded resolution (1° latitude × 1° longitude), as this was the best available resolution shared among datasets. Sea surface temperature (T; °C), salinity (S), nitrate (NO_3_−; μM), phosphate (PO_4_^3−^; μM), and silicic acid [Si(OH)_4_; μM] were obtained from World Ocean Atlas 2013 (1955–2012) fields (*34*, *43*, *48*). MLD (m) was included using the temperature criterion (*40*). Photosynthetically active radiation (PAR; μmol m^−2^ s^−1^) and chlorophyll (Chl; μg liter^−1^) were derived from the Sea-viewing Wide Field-of-view Sensor, using data from 1997 to 2007 (https://oceancolor.gsfc.nasa.gov). Sea surface wind stress (m s^−1^) was derived from the Cross-Calibrated Multi-Platform (*49*) using data from 1987 to 2011 (https://podaac.jpl.nasa.gov). Data on carbon dioxide partial pressure in the surface sea (pCO_2_; μatm) stem from (*50*).

We derived further predictors from these 10 variables: Photosynthetically available radiation over the MLD (MLPAR; μmol m^−2^ s^−1^) was computed from T, MLD, and Chl (*44*). We also used the excess concentration of NO_3_^−^, relative to the Redfield ratio of 16:1 (μM; N-star), computed as [NO_3_^−^] − 16[PO_4_^3−^]. The use of N-star, rather than NO_3_^−^, avoids strong global correlations between NO_3_^−^ and T (Spearman’s ρ = −0.71). Si-star, the ratio of [Si(OH)_4_] to [NO_3_^−^], was included as a predictor particularly relevant for the *Bacillariophyceae* (*45*). We also considered the temporal trends of T (dT/dt), NO_3_^−^ (dNO_3_^−^/dt), PO_4_^3−^ (dPO_4_^3−^/dt), Si(OH)_4_ [dSi(OH)_4_/dt], and MLD (dMLD/dt), calculated as the centered mean difference of the data of each month with its neighboring months. Logarithmic MLD, Chl, and nutrient levels to the base of 10 were used in addition to their original forms. Sea level height anomaly (m) (https://www.aviso.altimetry.fr/es/data/products/sea-surface-height-products/global/index.html) and nutricline depth (m), defined as the first depth at which nutrient levels exceeded a certain threshold (0.05 μM for NO_3_^−^ and 0.05:16 μM for PO_4_^3−^), were also tested, yet these two variables were discarded due to poor skill in single-predictor model tests. Net primary production (NPP; mg C m^−2^ day^−1^) was used for correlative analyses only, using data from 1998 to 2007 from the standard vertically generalized production model (http://www.science.oregonstate.edu/ocean.productivity).

### Species distribution models

SDMs fit statistical associations between species’ observed presences and environmental variables; i.e., they estimate a species’ realized ecological niche (*44*). SDMs provide a useful framework to explore large-scale distributions of phytoplankton species, based on two major assumptions: (i) Species are not dispersal limited in the open ocean (*51*, *52*) [but see (*53*)], a trait consistent with the generally wide geographic ranges of the species in our data; (ii) species are primarily controlled by environmental factors in their global distribution (*36*, *47*, *52*) and rapidly proliferate when conditions become suitable (*18*, *27*). Applying SDMs to study marine phytoplankton distributions has emerged relatively recently (*45*). Since distribution patterns of phytoplankton species might change seasonally (*30*), reflecting the generally short generational cycles of phytoplankton owing to their largely microbial nature, we used a monthly matchup between species’ presences and environmental variables to calibrate the niches. We then projected niches onto global environmental data fields at 1° and monthly resolution to obtain maps of species’ presence. We developed SDMs that address three principal sources of uncertainty: (i) biases in sampling effort, (ii) predictor selection, and (iii) algorithm choice. All steps used to build SDMs are described herein.

#### Data binning

We used phytoplankton presence data, rather than abundance data, as the former are less sensitive to differences in sampling methods and are more widely available. We binned species’ presence observations into monthly 1° latitude × 1° longitude resolution to match the resolution of environmental predictors. Multiple observations per species and 1° cell that stemmed from the same month, but potentially from different years, thus counted as a single presence, resulting in a total of 245,322 species presences. The monthly data binning may have removed signals of temporal changes in species’ distributions throughout the years. However, since data originated predominantly from a few decades between 1950 and 2000 (1984 ± 17; mean ± SD) and since climatic changes during this period were much smaller relative to current global amplitudes of environmental factors (for example, sea surface temperature spans ~ −1.8 to ~32°C) (*48*), we expect such changes to have only a minor impact on global SDM projections.

#### Environmental background data

Since absence data for phytoplankton are unreliable on the basis of traditional sampling methods (*20*) but required by our presence-absence SDMs, we selected background data (also termed pseudoabsences) for each species, using a so-called target-group approach (*54*). This approach addresses spatial and temporal sampling biases in field-based presence data of species via the selection of the number and location of pseudoabsences. We defined large groups of species as target groups, assuming that variation in sampling effort applied to the entire target group reflected variation in sampling applied to each species within the target group (*54*). The sampling of species’ background data from the target group served two purposes: (i) Background sampling followed a sampling scheme similar to that of the species’ presence data (and thus received similar bias), thereby balancing presence data bias when fitting SDMs; (ii) extensive ocean areas, which lacked sampling, were not misclassified as areas of species’ absences. We used the *Bacillariophyceae*, *Dinoflagellata*, and *Haptophyta* separately to define “group-specific target groups” for their constituent species, as these taxa had different global sampling schemes. For the remaining taxa, the number of species was insufficient to build group-specific target groups. For these taxa, we used the total species as the target group, excluding *Bacillariophyceae*, as presence data of the latter were strongly north-south imbalanced.

In parallel, we used the total species as target group to sample the background for each species, which we refer to as “total target group” approach. We found that richness results were robust to the use of total versus group-specific target groups (fig. S3B).

We sampled background data in a stratified manner from the target group, dividing both the T and MLD gradient (spanned by the target group) into nine equally spaced intervals, yielding 81 strata (T × MLD combinations). Sampling data from each stratum separately assured that the breadth of these two key environmental factors was reflected in the backgrounds of species. The target group’s presence data were gridded at monthly 1° resolution, before sampling backgrounds from it. We tested whether the density of these monthly 1° cells of the target group reflected original sampling efforts (approximated by the number of samples in the raw data) and found that the two measures were highly correlated (Spearman’s ρ = 0.94 for latitude; Spearman’s ρ = 0.99 for longitude; binning data at 1° latitude or 1° longitude, respectively). For each species, we sampled 10 times more background data than the species had presences (*55*). Within each of the 81 strata, background data were randomly sampled. The amount of background data sampled from a specific stratum was proportional to the number of monthly 1° cells provided by the target group in this stratum, thereby reflecting original sampling efforts.

#### Statistical complexity

Statistical algorithm choice represents a key source of uncertainty in SDMs (*56*). We constructed SDMs based on either GLM (using the R package *stats*), GAM (R package *mgcv*), or RF (R package *randomForest*), as three algorithms of increasing statistical response shape complexity (*57*). We considered the GAM as our standard algorithm because of its intermediate complexity. We used comparably few predictors (*n* = 4) in models and fitted simple response shapes to account for the relatively few presences of most phytoplankton species (*57*). GLM included linear and quadratic terms and a stepwise bidirectional predictor selection procedure. GAM used smoothing terms with five basis dimensions, estimated by penalized regression splines without penalization to zero for single variables. To equalize the overall weight of presences versus background data per species, background data in GAM and GLM were weighted by the ratio of species’ presence to background data points. RFs included 4000 trees, simple terms, and single end node size. The weighting of data in individual RF trees was balanced by randomly subsampling same amounts of background data as the species had presences.

#### Single predictor skill tests

In addition to algorithm choice, predictor choice represents a major source of uncertainty in phytoplankton SDMs, as these organisms are not well studied regarding their most important niche factors. To select powerful predictors for SDMs, we assessed the individual skill of an extensive number of candidate predictors (*n* = 25) in discriminating species’ presences versus background data. The results of this test also served to identify the key environmental drivers of species’ distributions independently of the SDM analysis. We fitted single-factor GLM, GAM, and RF models to the presences versus background data of each species, for each candidate predictor. The species (*n* = 567) considered for predictor analyses generally contained a minimum of 24 presences as was used as a lower threshold for species in SDMs. Model explanatory skill was evaluated using the adjusted *D*^2^ (for GLM and GAM) and the out-of-bag error (for RF) statistic. For each species, predictors were ranked according to these statistics, and the mean variable ranks obtained across GLM, GAM, and RF served as a basis for predictor selection. We performed several sensitivity tests to evaluate the robustness of the predictor ranking. We compared rarely versus more frequently sampled species (i.e., ≥15, ≥24, and ≥50 presences), used different variables for the stratification of background sampling, and applied spatial thinning of species’ presences to a distance ≥300 or ≥600 km (using the R package *spThin*), which reduces potentially confounding effects of autocorrelation. None of these modifications changed the result that temperature was the top-ranked predictor across total species. However, the rank of predictors other than temperature tended to vary between setups.

#### Predictor choice for models

To capture predictor-based uncertainties, we fitted five member models, each using a different set of four predictors, for building an SDM ensemble. The species (*n* = 567) considered for modeling contained ≥24 presences, which corresponds to a presence-to-predictor ratio ≥6 per species. We used a randomization approach to select the four predictors per member model, using the test-based predictor ranking (see above) of each species as a basis. For the first member model, we selected four predictors at random, without replacement from those predictors that ranked among the 10 most powerful predictors per species. We omitted Spearman’s rank correlations between predictors greater than |0.7| in each predictor set (computed from the predictor data at global monthly 1° resolution). Predictors of the four other members were composed by the same criterion. Yet, we allowed each predictor to be selected only up to twice among the five members to omit biases due to overrepresentation of individual predictors in SDMs. If sampling among the top 10 predictors did not provide a sufficient number of predictors for the 5 sets × 4 predictors (given the correlation criterion), candidate predictors that ranked >10 were selected. Predictors were equally used in GAM, GLM, and RF.

#### Monte Carlo simulation

We used a Monte Carlo simulation to quantify uncertainty in our results emerging from the choice of different predictor sets. For each species modeled, we randomly selected one of the five predictor sets prepared (see above) to fit the SDM of the species and then calculated richness and turnover of total species. This procedure was repeated (*n* = 1000 runs) and we present the SD across the runs (Figs. 1, C and D, and 3B).

#### Evaluation of model members and ensemble construction

For each species, we evaluated the predictive skill of each member model based on a repeated (4×) split-sample cross-validation test. In this test, the species’ presences and background data are randomly split into four parts. The SDM member model is iteratively trained on the basis of three parts (75%) of the data and used to predict the remaining one-fourth (25%) of the data. The predicted values are then compared against the true values. We calculated the true skill statistic (TSS) (*58*) of this test. TSS ranges from −1 to +1 with values greater than zero indicating models performing better than at random. We retained member models with a TSS score of at least 0.35 for the construction of our SDM ensembles. Successful member models were then projected globally onto monthly (*n* = 12 months) environmental data fields, yielding probabilistic maps of species’ presence. Presence probabilities were generally higher for high-latitude species than for lower-latitude species, as ecological niches at high latitudes were readily captured by SDMs. To avoid spatial biases in multispecies analyses, we therefore binarized the projected probabilities to presence-absence from thresholds maximizing the TSS (package *presenceAbsence*). For each month, we averaged the successful member models of each particular species to obtain monthly ensemble mean projections. Each species thus obtained a value between zero and one per monthly 1° cell. We did not further binarize the ensemble projections to presence-absence, as binarization tends to overestimate the species’ presence toward the edges of the projected presence area, relative to its center. We hence argue that our ensemble mean projections characterize species’ distribution patterns at a higher level of detail compared to 0/1 projections and are better suited for multispecies analyses, in line with previous work showing that the sum of overlapping 0/1 projections tended to overestimate species richness (*59*).

### Global species richness map

We used the summation of monthly SDM ensemble projections of the successfully modeled species (536 species for GAM, 529 species for GLM, and 538 species for RF) to obtain 12 monthly estimates of phytoplankton species richness (data file S1). The global species richness map (Fig. 1A) represents the average of these monthly estimates. We examined the robustness of the global species richness pattern with respect to balancing the relative representation of major taxa in our model analysis (fig. S3A). For each major taxon (table S4), we divided the number of species included in SDMs by the number of its totally known species, and we weighted the individual species inversely to this ratio in the corrected global richness pattern (fig. S3A).

### Global species turnover map

Species turnover is a measure of the difference in species composition between two ecological communities. We assessed species turnover through time, analyzing the fraction of species whose identities changed between months (“species replacement”). We derived species communities at monthly 1° resolution from the species’ monthly ensemble mean projections (for each monthly 1° cell, species with values larger than 0.5 counted as present). We calculated species turnover at 1° resolution between consecutive months (*n* = 12 monthly pairs) and averaged the results of the monthly pairs over the full year to obtain the species turnover map (Fig. 1B). We used the turnover component of the Jaccard dissimilarity as implemented in R’s *betapart* package.

### Explanatory power of single variables for global richness patterns

We tested whether richness itself could be modeled successfully using environmental variables. We fitted species richness using GLMs with a single predictor, thus assessing their univariate predictive skill (table S2). We fitted GLMs both to the species richness obtained from SDMs (aggregated from GAM, at monthly 1° resolution) and to the species richness observed at a sample level in the raw data (table S2). Each local sample was defined by a unique combination of latitude, longitude, depth, year, month, and day of collection in the phytoplankton raw data. Environmental variables were matched with richness values at 1° resolution, using monthly climatological data. The quality of linear model fits between single variables and GAM-based richness (at monthly 1° resolution) presented in the text is indicated by the *R*^2^ statistic.

### Global richness patterns in aggregate observational data

The number of detected species varied strongly between samples (fig. S1, B and C). To infer robust species richness patterns directly from raw observations (figs. S4 and S5), we split raw data into coarse environmental or geographic strata and repeatedly selected a certain number of samples at random, from each stratum. Determining the integral richness from the pooled samples, per stratum, served to depict richness patterns in the observational data. This approach, in part, ameliorates the problem of severe undersampling of richness by traditionally small sampling volumes (*20*).

### Sensitivity of richness patterns to species detection

Our analysis included an extensive number of phytoplankton species and spanned all major phytoplankton taxa (table S4). However, results are limited to species for which in situ data were available. Patterns thus reflect the subset of detectable, and likely common, species, while potentially overlooking an unknown number of low-abundance species. We tested whether richness patterns in our data were robust to the omission of species that were relatively more rarely observed (figs. S4 and S5). We found that patterns were largely robust to their exclusion, in line with previous findings showing that global richness patterns are best approximated by subsets of common (rather than by rare) species (*60*).

### Species range analysis

Species’ observed ranges for key environmental factors were defined by the maximum and minimum value obtained from matching up the species’ raw observations with sea surface temperature (*48*), sea surface wind stress (*49*), MLD (*40*), and additional factors, at monthly 1° resolution (Fig. 2B and fig. S6). Species’ latitudinal ranges corresponded to the maximum and minimum latitude of raw observations (Fig. 2C). We assessed the expected maximum species richness over the full range of each environmental variable in the ocean based on species’ observed presence ranges. At each environmental value (Fig. 2, B and C, and fig. S6), maximum richness is defined as the sum of the species’ ranges that overlapped, and range size is defined as the species’ mean range size.

To denote parts of the environmental gradients where edge effects likely distorted species’ observed range patterns, we created a null model of the environmental response in expected maximum richness and average range size. To this end, we randomized the species’ presences in the observational data (*n* = 100 runs) and derived species’ range limits and expected richness/mean range size based on these randomized data. Thus, our null model represents the expected maximum richness/mean range size in absence of any environmental sorting of species’ ranges. We identified areas with likely edge effects as those areas where the null model values deviated significantly (using the 95% confidence interval range) from the null model value obtained in the center (i.e., not affected by edge effects) of the environmental gradient studied. In Fig. 2 (B and C) and fig. S6, we only show estimates of maximum richness (red line) and range size (blue line) for areas unaffected by edge effects.

## Supplementary Material

http://advances.sciencemag.org/cgi/content/full/5/5/eaau6253/DC1

Download PDF

Data file S1

## SUPPLEMENTARY MATERIALS

Supplementary material for this article is available at http://advances.sciencemag.org/cgi/content/full/5/5/eaau6253/DC1 Supplementary Materials and Methods Fig. S1. Distribution of phytoplankton presence observations in space and time. Fig. S2. SDM performance for the three statistical algorithms used. Fig. S3. Sensitivity of global species richness patterns to methodological choices. Fig. S4. Latitudinal species richness gradients derived from the observational raw data. Fig. S5. Species richness–temperature relationships derived from the observational raw data. Fig. S6. Species ranges for key environmental factors. Table S1. Fraction of equatorial species recorded at higher latitudes. Table S2. Single variable model skill for predicting species distributions and global richness. Table S3. Contribution of sources to the phytoplankton dataset. Table S4. Statistics on data collected and species modeled within major taxon groups. Data file S1. Monthly species richness diagnosed at global scale, 1° spatial resolution. References (*61*–*65*)
