# Supplementary material for: Global pattern of phytoplankton diversity driven by temperature and environmental variability
Source: Sci Adv. 2019 May 15;5(5):eaau6253. doi: 10.1126/sciadv.aau6253 (PMC6520023; doi:10.1126/sciadv.aau6253)
Supplement: http://advances.sciencemag.org/cgi/content/full/5/5/eaau6253/DC1 [file supp_5_5_eaau6253__index.html]

Science Advances | Science Advances

## Supplementary Materials

**The PDF file includes:**

- Supplementary Materials and Methods
- Fig. S1. Distribution of phytoplankton presence observations in space and time.
- Fig. S2. SDM performance for the three statistical algorithms used.
- Fig. S3. Sensitivity of global species richness patterns to methodological choices.
- Fig. S4. Latitudinal species richness gradients derived from the observational raw data.
- Fig. S5. Species richness–temperature relationships derived from the observational raw data.
- Fig. S6. Species ranges for key environmental factors.
- Table S1. Fraction of equatorial species recorded at higher latitudes.
- Table S2. Single variable model skill for predicting species distributions and global richness.
- Table S3. Contribution of sources to the phytoplankton dataset.
- Table S4. Statistics on data collected and species modeled within major taxon groups.
- References (*61*–*65*)

Download PDF

**Other Supplementary Material for this manuscript includes the following:**

- Data file S1 (.nc format). Monthly species richness diagnosed at global scale, 1° spatial resolution.

**Files in this Data Supplement:**

- Adobe PDF - aau6253\_SM.pdf
