# Supplementary material for: Global pattern of phytoplankton diversity driven by temperature and environmental variability
Source: Sci Adv. 2019 May 15;5(5):eaau6253. doi: 10.1126/sciadv.aau6253 (PMC6520023; doi:10.1126/sciadv.aau6253)
Supplement: Download PDF [file aau6253_SM.pdf]

Science Advances
AAAS

advances.sciencemag.org/cgi/content/full/5/5/eaau6253/DC1

# Supplementary Materials for

## Global pattern of phytoplankton diversity driven by temperature and environmental variability

Damiano Righetti*, Meike Vogt, Nicolas Gruber, Achilleas Psomas, Niklaus E. Zimmermann

*Corresponding author. Email: damiano.righetti@env.ethz.ch

Published 15 May 2019, *Sci. Adv.* **5**, eaau6253 (2019)
DOI: 10.1126/sciadv.aau6253

**The PDF file includes:**

Supplementary Materials and Methods
Fig. S1. Distribution of phytoplankton presence observations in space and time.
Fig. S2. SDM performance for the three statistical algorithms used.
Fig. S3. Sensitivity of global species richness patterns to methodological choices.
Fig. S4. Latitudinal species richness gradients derived from the observational raw data.
Fig. S5. Species richness–temperature relationships derived from the observational raw data.
Fig. S6. Species ranges for key environmental factors.
Table S1. Fraction of equatorial species recorded at higher latitudes.
Table S2. Single variable model skill for predicting species distributions and global richness.
Table S3. Contribution of sources to the phytoplankton dataset.
Table S4. Statistics on data collected and species modeled within major taxon groups.
References (*61*–*65*)

**Other Supplementary Material for this manuscript includes the following:**

(available at advances.sciencemag.org/cgi/content/full/5/5/eaau6253/DC1)

Data file S1 (.nc format). Monthly species richness diagnosed at global scale, 1° spatial resolution.

## Supplementary Materials and Methods

### 1.Taxonomic Reference Lists and Digital Data Access

Phytoplankton presence data from OBIS (https://www.obis.org) were accessed using the package *robis* in the R statistical language (*61*). Data were queried using the species names obtained from the taxonomic reference list of OBIS, which was accessed on 4 December 2015, using the R packages *RPostgreSQL* and *devtools*. This taxonomic reference list followed the World Register of Marine Species (WoRMS; http://www.marinespecies.org). Data from GBIF (https://www.gbif.org) were queried using the R package *rgbif* and the taxonomic reference list of GBIF (*62*). Higher rank taxonomy in this list followed the Catalogue of Life (http://www.catalogueoflife.org).

### 2. Taxonomic Harmonization

We validated a total of 2441 species names in the raw data against the taxonomic reference list of Algaebase (*63*) (http://www.algaebase.org), an extensive digital database with emphasis on algae taxonomy. The species names were subsequently cleaned or corrected following taxonomic expert opinion (M. Guiry, personal communication, August 2017). Expert validation led to the exclusion of 503 species names that could not be traced to any taxonomic entity on Algaebase, which was either accepted or preliminarily accepted, leaving 1938 species names. Spelling variants and synonymous names were merged, resulting in 1677 species names.

### 3. Selection of Extant Marine Taxa

We next excluded species that were classified as 'fossil only' or 'fossil' based on either Algaebase or the World Register of Marine Species (WoRMS; http://www.marinespecies.org, accessed August 2017). If the species belonged to genera with fossil types, these species were excluded if they had no habitat entry on both Algaebase and WoRMS. Species with a habitat entry and uniquely classified as 'freshwater' on both Algaebase and WoRMS were excluded from analyses. However, the following species classified as 'freshwater' were retained, as they received at least 24 observations from the open ocean and were thus assumed to be marine: *Aulacoseira granulata*, *Chaetoceros wighamii, Diatoma rhombica, Dinobryon balticum, Gymnodinium wulffii, Tripos candelabrum, Tripos euarcuatus.* The resulting list contained 1451 species. Subsequent exclusion of species without any observations within the oceanic surface mixed-layer (*40*) resulted in 1300 remaining species. Using a minimum threshold of 24 gridded presences (at monthly 1°-resolution) per species, a total of 567 species were considered for subsequent modeling, among which 553 species (listed below) could be modeled by any of the three statistical algorithms with reasonable predictive skill (i.e., TSS ≥0.35).

### 4. List of Taxa Examined by Species Distribution Models

*Bacillariophyceae: Achnanthes longipes, Actinocyclus curvatulus, Actinocyclus octonarius, Actinoptychus octonarius, Actinoptychus senarius, Actinoptychus splendens, Amphiprora gigantean, Asterionellopsis glacialis, Asterolampra marylandica, Asteromphalus brookei, Asteromphalus cleveanus, Asteromphalus flabellatus, Asteromphalus heptactis, Asteromphalus hyalinus, Asteromphalus parvulus, Asteromphalus robustus, Asteroplanus karianus, Attheya septentrionalis, Aulacoseira granulate, Azpeitia nodulifera, Bacillaria paxillifera*[GAMg,GLMg]*, Bacteriastrum comosum, Bacteriastrum delicatulum, Bacteriastrum elegans, Bacteriastrum elongatum, Bacteriastrum furcatum, Bacteriastrum hyalinum, Bacteriastrum mediterraneum, Bacterosira bathyomphala, Bellerochea horologicalis*[GAMg,GLMg,RFg,GAMt]*, Bellerochea malleus, Biddulphia alternans, Cerataulina pelagica, Chaetoceros aequatorialis, Chaetoceros affinis, Chaetoceros anastomosans, Chaetoceros atlanticus, Chaetoceros bacteriastroides, Chaetoceros borealis, Chaetoceros brevis, Chaetoceros bulbosus, Chaetoceros castracanei, Chaetoceros cinctus, Chaetoceros coarctatus, Chaetoceros compressus, Chaetoceros concavicornis, Chaetoceros constrictus, Chaetoceros convolutes, Chaetoceros costatus, Chaetoceros criophilus, Chaetoceros curvisetus, Chaetoceros dadayi, Chaetoceros danicus*[GLMt]*, Chaetoceros debilis, Chaetoceros decipiens, Chaetoceros densus, Chaetoceros diadema, Chaetoceros dichaeta, Chaetoceros didymus, Chaetoceros diversus, Chaetoceros eibenii, Chaetoceros furcellatus, Chaetoceros gracilis, Chaetoceros hyalochaetae, Chaetoceros indicus, Chaetoceros laciniosus, Chaetoceros laevis, Chaetoceros lauderi, Chaetoceros lorenzianus, Chaetoceros messanensis, Chaetoceros neglectus, Chaetoceros pacificus, Chaetoceros paradoxus, Chaetoceros pelagicus, Chaetoceros pendulus, Chaetoceros peruvianus, Chaetoceros phaeoceros, Chaetoceros pseudocrinitus, Chaetoceros pseudocurvisetus, Chaetoceros pseudodichaetus, Chaetoceros radicans, Chaetoceros rostratus,*

*Chaetoceros saltans, Chaetoceros seiracanthus, Chaetoceros seriacanthus, Chaetoceros seychellarum, Chaetoceros similis, Chaetoceros simplex, Chaetoceros socialis, Chaetoceros subsecundus, Chaetoceros teres, Chaetoceros tetrastichon, Chaetoceros tortissimus, Chaetoceros vanheurckii, Chaetoceros wighamii, Chaetoceros willei, Climacodium biconcavum, Climacodium frauenfeldianum, Climacosphenia moniligera, Corethron hystrix, Corethron pennatum*[GLMg,RFg]*, Coscinodiscus antarcticus, Coscinodiscus asteromphalus, Coscinodiscus centralis, Coscinodiscus concinnus, Coscinodiscus curvatulus, Coscinodiscus gigas, Coscinodiscus granii, Coscinodiscus marginatus, Coscinodiscus oculus-iridis, Coscinodiscus radiates, Coscinodiscus subbulliens, Coscinodiscus wailesii, Cylindrotheca fusiformis, Dactyliosolen antarcticus, Dactyliosolen fragilissimus, Detonula confervacea, Detonula pumila, Diatoma rhombica, Ditylum brightwellii, Ditylum sol, Entomoneis alata, Entomoneis paludosa*[GLMg,GLMt]*, Ephemera planamembranacea, Ethmodiscus gazellae, Eucampia Antarctica, Eucampia cornuta, Eucampia zodiacus, Eucampia zoodiacus, Eupyxidicula turris, Fragilaria striatula, Fragilariopsis curta, Fragilariopsis cylindrus, Fragilariopsis doliolus, Fragilariopsis kerguelensis, Fragilariopsis obliquecostata, Fragilariopsis oceanica, Gossleriella tropica, Grammatophora angulosa, Grammatophora marina, Grammatophora oceanica, Guinardia cylindrus, Guinardia delicatula, Guinardia flaccida, Guinardia striata*[GLMg,GLMt]*, Haslea wawrikae, Helicotheca tamesis, Hemiaulus chinensis, Hemiaulus hauckii, Hemiaulus membranaceus, Hemidiscus cuneiformis, Lauderia annulata, Leptocylindrus danicus, Leptocylindrus minimus, Licmophora abbreviate, Licmophora lyngbyei, Lioloma delicatulum, Lioloma pacificum, Lithodesmium undulatum, Mastogloia rostrata, Melosira borreri, Melosira moniliformis, Membraneis challenger, Meuniera membranacea, Navicula pelagica, Neocalyptrella robusta, Nitzschia bicapitata*[GAMt,GLMt]*, Nitzschia bilobata, Nitzschia frigida, Nitzschia longissima, Nitzschia pacifica, Nitzschia sicula, Nitzschia sigma, Nitzschia tenuirostris, Nitzschia vitrea, Odontella aurita, Odontella longicruris, Plagiotropis lepidoptera, Planktoniella sol, Pleurosigma angulatum, Pleurosigma directum, Pleurosigma diversestriatum, Pleurosigma elongatum, Pleurosigma nicobaricum, Pleurosigma normanii, Pleurosigma simonsenii, Podosira stelligera, Porosira glacialis, Proboscia indica, Proboscia inermis, Proboscia truncate, Pseudo-nitzschia lineola, Pseudo-nitzschia pseudodelicatissima, Pseudo-nitzschia pungens, Pseudo-nitzschia subfraudulenta, Pseudosolenia calcar-avis, Rhabdonema arcuatum, Rhaphoneis amphiceros*[GAMg,GLMg,GLMt]*, Rhizosolenia acuminate, Rhizosolenia bergonii, Rhizosolenia castracanei, Rhizosolenia chunii, Rhizosolenia clevei, Rhizosolenia formosa, Rhizosolenia hyaline, Rhizosolenia imbricata*[GAMg,GLMg,RFg,GAMt,GLMt]*, Rhizosolenia setigera, Rhizosolenia temperei, Roperia tesselata, Shionodiscus gracilis, Shionodiscus oestrupii, Skeletonema costatum*[GAMg,GLMg,RFg,GAMt,GLMt]*, Stellarima stellaris, Stephanopyxis nipponica, Stephanopyxis palmeriana, Striatella unipunctata, Thalassionema bacillare, Thalassionema frauenfeldii, Thalassiosira aestivalis, Thalassiosira angulate, Thalassiosira angustelineata, Thalassiosira antarctica, Thalassiosira decipiens, Thalassiosira eccentric, Thalassiosira gracilis, Thalassiosira gravida, Thalassiosira hyaline, Thalassiosira leptopus, Thalassiosira mendiolana, Thalassiosira minima, Thalassiosira nordenskioeldii, Thalassiosira partheneia, Thalassiosira subtilis, Thalassiothrix heteromorpha, Trieres chinensis, Trieres mobiliensis, Trieres regia*

*Chlorophyta: Halosphaera viridis, Pterosperma moebii, Pterosperma vanhoeffenii, Ulva fasciata*

*Chrysophyceae: Dictyocha fibula, Dinobryon balticum, Octactis speculum*

*Cryptophyta: Hillea fusiformis, Leucocryptos marina*

*Cyanobacteria: Prochlorococcus, Synechococcus, Trichodesmium erythraeum, Trichodesmium thiebautii*

*Dinoflagellata: Akashiwo sanguinea, Alexandrium monilatum, Amphidinium acutissimum, Amphidinium carterae, Amphidinium sphenoides, Amphisolenia bidentata, Amphisolenia bispinosa, Archaeperidinium minutum, Azadinium caudatum, Bitectatodinium spongium, Blepharocysta splendor-maris, Ceratium arcticum, Ceratium breve, Ceratium contrarium, Ceratium falcatiforme, Ceratium falcatum, Ceratium gibberum, Ceratium gravidum, Ceratium horridum*[GAMg,GLMg,RFg]*, Ceratium longirostrum, Ceratium massiliense, Ceratium pavillardii, Ceratium setaceum, Ceratium symmetricum, Ceratium trichoceros, Ceratocorys armata, Ceratocorys bipes, Ceratocorys horrida, Ceratocorys reticulate, Ceratoperidinium falcatum, Cladopyxis brachiolata, Cochlodinium pupa, Cochlodinium vinctum, Corythodinium tesselatum, Dinophysis acuminate, Dinophysis acuta, Dinophysis apicata, Dinophysis argus, Dinophysis caudate, Dinophysis fortii, Dinophysis hastate, Dinophysis norvegica, Dinophysis ovum, Dinophysis parvula, Dinophysis porodictyum, Dinophysis sacculus, Dinophysis schroederi, Dinophysis schuettii, Dinophysis tripos, Diplopelta asymmetrica, Diplopelta steinii, Diplopsalis lenticular, Diplopsalopsis bomba, Echinidinium delicatum, Goniodoma sphaericum, Gonyaulax birostris, Gonyaulax diegensis, Gonyaulax digitalis, Gonyaulax elongate, Gonyaulax membranacea, Gonyaulax monacantha, Gonyaulax pacifica, Gonyaulax polygramma, Gonyaulax scrippsae, Gonyaulax spinifera, Gymnodinium agiliforme, Gymnodinium arcticum, Gymnodinium aureolum, Gymnodinium catenatum, Gymnodinium gracile, Gymnodinium marinum, Gymnodinium simplex, Gymnodinium uberrimum, Gymnodinium wulffii, Gyrodinium flagellare, Gyrodinium fusiforme, Gyrodinium pingue, Gyrodinium prunus, Gyrodinium spirale, Gyrodinium wulffii, Heterocapsa niei, Heterocapsa rotundata, Heterocapsa triquetra, Heterodinium blackmanii. Impagidinium aculeatum, Impagidinium patulum, Impagidinium sphaericum, Kapelodinium vestifici, Karenia brevis, Karenia mikimotoi, Karlodinium veneficum, Kofoidinium velleloides, Lebessphaera urania, Lebouridinium glaucum. Leonella granifera, Lingulodinium polyedra, Mesoporos perforatus, Neoceratium breve, Neoceratium hexacanthum, Ornithocercus heteroporus, Ornithocercus magnificus, Ornithocercus quadratus, Ornithocercus splendidus,*

*Ornithocercus steinii, Ornithocercus thumii, Ornithocercus thurnii, Oxytoxum caudatum, Oxytoxum constrictum, Oxytoxum curvatum, Oxytoxum elegans, Oxytoxum gracile, Oxytoxum laticeps, Oxytoxum longiceps, Oxytoxum milneri, Oxytoxum parvum, Oxytoxum reticulatum, Oxytoxum sceptrum, Oxytoxum scolopax, Oxytoxum sphaeroideum, Oxytoxum tesselatum, Oxytoxum turbo, Oxytoxum variabile, Pentapharsodinium dalei, Peridinium breve, Phalacroma doryphorum, Phalacroma favus, Phalacroma lens, Phalacroma mitra, Phalacroma oxytoxoides, Phalacroma rapa, Phalacroma rotundatum, Podolampas bipes, Podolampas elegans, Podolampas palmipes, Podolampas spinifera, Polykrikos schwartzii, Preperidinium meunieri, Pronoctiluca pelagica, Pronoctiluca spinifera, Prorocentrum arcuatum, Prorocentrum balticum, Prorocentrum cordatum, Prorocentrum dentatum, Prorocentrum gracile, Prorocentrum lima, Prorocentrum mexicanum, Prorocentrum micans, Prorocentrum rostratum, Prorocentrum scutellum, Prorocentrum triestinum, Protoceratium reticulatum*[GLMt]*, Protoperidinium abei, Protoperidinium americanum, Protoperidinium bipes, Protoperidinium brevipes, Protoperidinium brochii, Protoperidinium cerasus, Protoperidinium claudicans, Protoperidinium conicoides, Protoperidinium crassipes, Protoperidinium curtipes, Protoperidinium defectum, Protoperidinium depressum, Protoperidinium diabolus, Protoperidinium divergens, Protoperidinium elegans, Protoperidinium excentricum, Protoperidinium fatulipes, Protoperidinium grahamii, Protoperidinium grande, Protoperidinium granii, Protoperidinium latidorsale, Protoperidinium latispinum, Protoperidinium leonis, Protoperidinium longipes, Protoperidinium longispinum, Protoperidinium mediterraneum, Protoperidinium mendiolae, Protoperidinium mite, Protoperidinium oblongum, Protoperidinium obtusum, Protoperidinium oceanicum, Protoperidinium ovatum, Protoperidinium ovum, Protoperidinium pallidum, Protoperidinium pedunculatum, Protoperidinium pellucidum, Protoperidinium pentagonum, Protoperidinium peruvianum, Protoperidinium punctulatum, Protoperidinium pyriforme, Protoperidinium pyrum, Protoperidinium quarnerense, Protoperidinium steinii, Protoperidinium stellatum, Protoperidinium subinerme, Protoperidinium tenuissimum, Protoperidinium tristylum, Protoperidinium tuba, Protoperidinium venustum, Ptychodiscus noctiluca, Pyrocystis elegans, Pyrocystis fusiformis, Pyrocystis hamulus, Pyrocystis lunula, Pyrocystis robusta, Pyrophacus horologium, Pyrophacus steinii, Pyrophacus vancampoae, Schuettiella mitra, Scrippsiella acuminate, Scrippsiella regalis, Spiniferites pachydermus, Spiraulax kofoidii, Thoracosphaera heimii, Torodinium robustum, Triadinium polyedricum, Trinovantidinium applanatum, Tripos arietinus*[GAMg,GLMg]*, Tripos azoricus, Tripos balechii, Tripos belone, Tripos bigelowii, Tripos bucephalus, Tripos buceros, Tripos candelabrum, Tripos carnegiei, Tripos carriensis, Tripos compressus, Tripos concilians, Tripos contortus, Tripos declinatus, Tripos deflexus, Tripos dens, Tripos digitatus, Tripos euarcuatus, Tripos eugrammus, Tripos extensus, Tripos furca*[GAMg,GLMg,RFg,RFt]*, Tripos geniculatus, Tripos gravidus, Tripos hexacanthus, Tripos incises, Tripos inflatus, Tripos karstenii, Tripos kofoidii, Tripos lamellicornis, Tripos limulus, Tripos lineatus*[GAMg,GLMg,RFg]*, Tripos longipes*[GAMg,GLMg,RFg]*, Tripos lunula, Tripos macroceros*[GAMg,GLMg,RFg,RFt]*, Tripos massiliensis, Tripos minutus*[GLMg,RFg]*, Tripos paradoxides, Tripos pentagonus, Tripos platycornis, Tripos pulchellus, Tripos ranipes, Tripos strictus, Tripos teres, Tripos trichoceros, Tripos vulture, Tryblionella compressa*[GAMt,GLMt]

*Euglenoidea: Eutreptiella gymnastica*

*Haptophyta: Acanthoica acanthifera, Acanthoica quattrospina*[GAMg,GLMg]*, Algirosphaera robusta, Calcidiscus leptoporus*[GAMg,GLMg,RFg]*, Calciopappus rigidus, Calciosolenia brasiliensis, Calciosolenia granii, Calciosolenia murrayi, Coccolithus pelagicus*[GAMg,GLMg,RFg,GLMt]*, Coronosphaera mediterranea, Discosphaera tubifera, Emiliania huxleyi*[GAMg,GLMg,RFg]*, Florisphaera profunda, Gephyrocapsa caribbeanica, Gephyrocapsa ericsonii, Gephyrocapsa muellerae, Gladiolithus flabellatus, Hayaster perplexus, Helicosphaera carteri*[GAMg,GLMg,RFg]*, Helladosphaera cornifera, Michaelsarsia adriatica, Michaelsarsia elegans, Oolithotus fragilis*[GLMg]*, Ophiaster hydroideus, Phaeocystis antarctica, Phaeocystis pouchetii, Reticulofenestra sessilis, Rhabdolithes claviger*[GLMg]*, Rhabdosphaera hispida, Rhabdosphaera xiphos, Syracosphaera molischii, Syracosphaera nodosa, Syracosphaera prolongata, Syracosphaera pulchra*[GAMg,GLMg,RFg]*, Umbellosphaera irregularis, Umbellosphaera tenuis, Umbilicosphaera hulburtiana, Umbilicosphaera sibogae*[GLMg]

The geographic distribution of 553 species (listed above) was successfully modeled by any of the three statistical algorithms used times any of the two background selection techniques used (Materials and Methods). Superscripts indicate particular combinations of algorithm and background choice in which individual species were not successfully modeled: GAM, generalized additive models; GLM, generalized linear models; RF, random forest; g, group specific target-group background; t, total target-group background. 536 species were successfully modeled (i.e., TSS ≥0.35) by GAM with group-specific target groups.

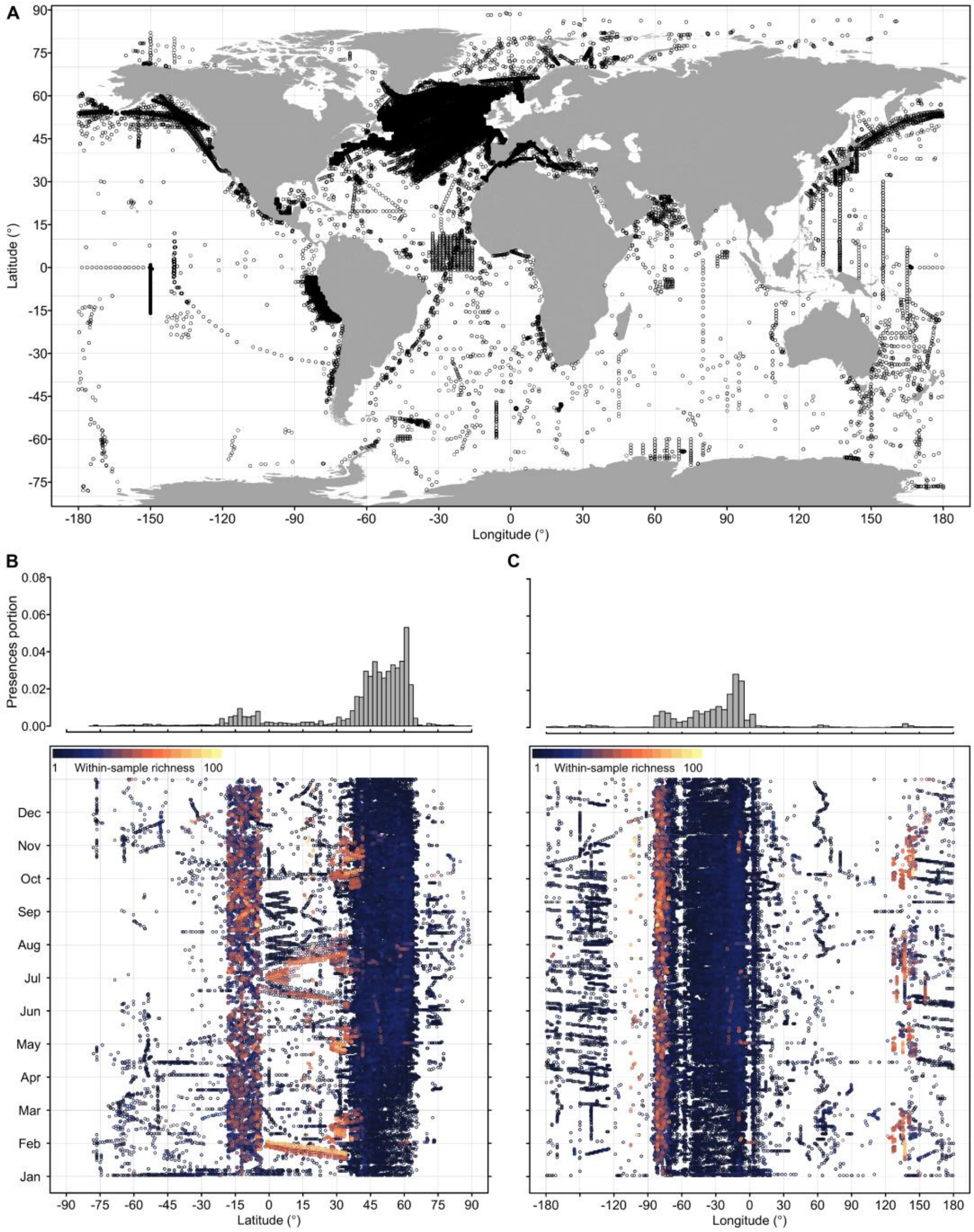


**Fig. S1. Distribution of phytoplankton presence observations in space and time.** (**A**) Black circles denote the positions of *in situ* observations ($n$ = 1,056,363) used in this study. The observations are also plotted as a function of climatological month and (**B**) latitude or (**C**) longitude. Associated histograms indicate the relative frequency of presence observations along latitude (B) and longitude (C). Colors illustrate the number of species detected within individual samples (Materials and Methods).

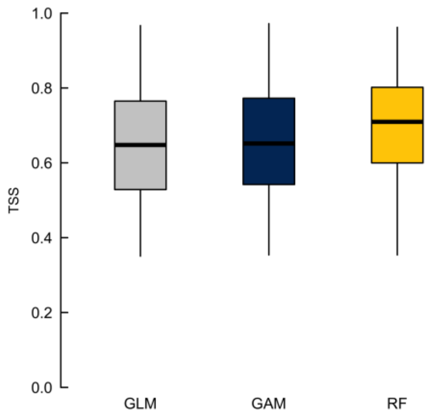


**Fig. S2. SDM performance for the three statistical algorithms used.** Box-whisker plots display the average (thick horizontal lines) true skill statistic (TSS) of all models fitted to species observations, which were either calibrated using generalized additive models (GAM), generalized linear models (GLM) or random forest models (RF). The models shown had a minimum TSS score of 0.35, and were included in analyses of species richness. The number of species obtaining a TSS equal or larger than 0.35 was 536 for GAM, 529 for GLM, and 538 for RF. Boxes indicate the first and third quartiles for TSS distribution around the mean and whiskers denote 1.5 times the inter-quartile range.

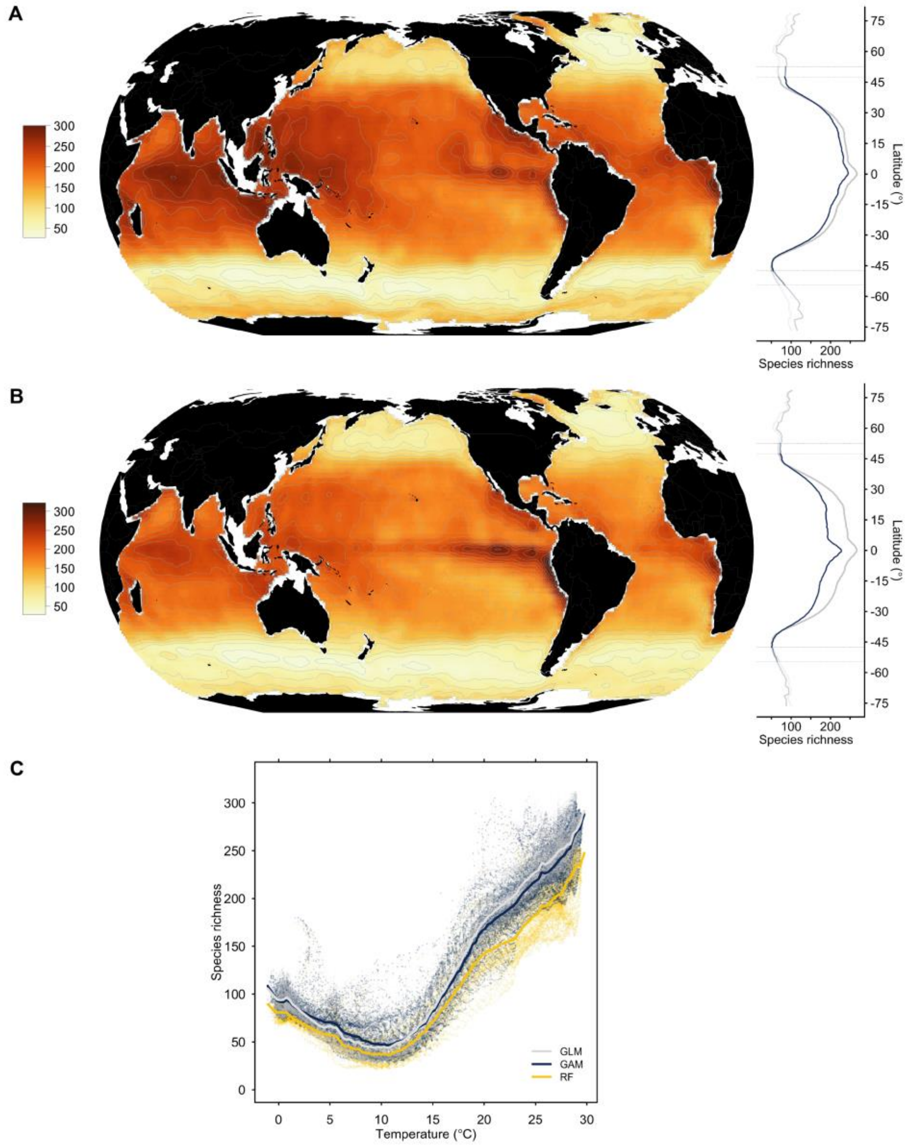


**Fig. S3. Sensitivity of global species richness patterns to methodological choices.** (**A**) Global map of phytoplankton species richness as shown in Fig. 1A (*n* = 536 species), but applying a weighting factor to individual species. The weighting factor corrected for the relative representation of major phytoplankton taxa in our analysis, using the proportion of species of a specific taxon captured in our SDMs (table S4; Materials and Methods). (**B**) Global map as shown in Fig. 1A, but modeling individual species based on background data from the total target-group, rather than group-specific target groups (Materials and Methods). Latitudinal richness gradients are shown for each map, fitting the means per degree latitude (dark blue lines). Grey lines indicate the GAM-based result from Fig. 1C. (**C**) Relationships between the annual mean of monthly phytoplankton species richness and temperature based on the three different algorithms used. Colored lines indicate the mean trend for each algorithm (regressions with local polynomial fitting).

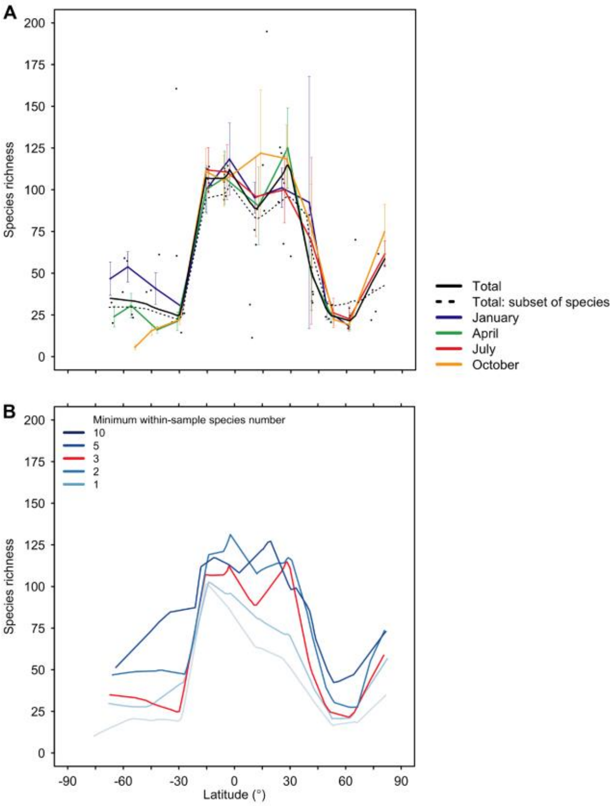


**Fig. S4. Latitudinal species richness gradients derived from the observational raw data**. (**A**) Colored lines show the species richness gradients for individual months, using a resampling procedure with equally spaced latitudinal intervals (size of ~14° latitude; *n* = 12 intervals). Phytoplankton species richness was obtained by summing up species from ten samples selected at random per interval and month (*n* = 12 months). Dots show the mean richness, and error bars indicate the standard deviations from the means across 1000 resampling runs. The mean trends for four individual months are highlighted in color. The solid black line shows the annual mean trend across all months (regression with local polynomial fitting) using total species (*n* = 1300) and the dotted black line shows the mean trend for the subset of the relatively more abundant species also included in SDM analyses (*n* = 536; GAM). (**B**) The red line indicates the mean trend as for (A). Blue lines show how this trend changes depending on the minimum quality criterion used for samples to be retained in the analysis (i.e. different thresholds with respect to the minimum species number detected per sample).

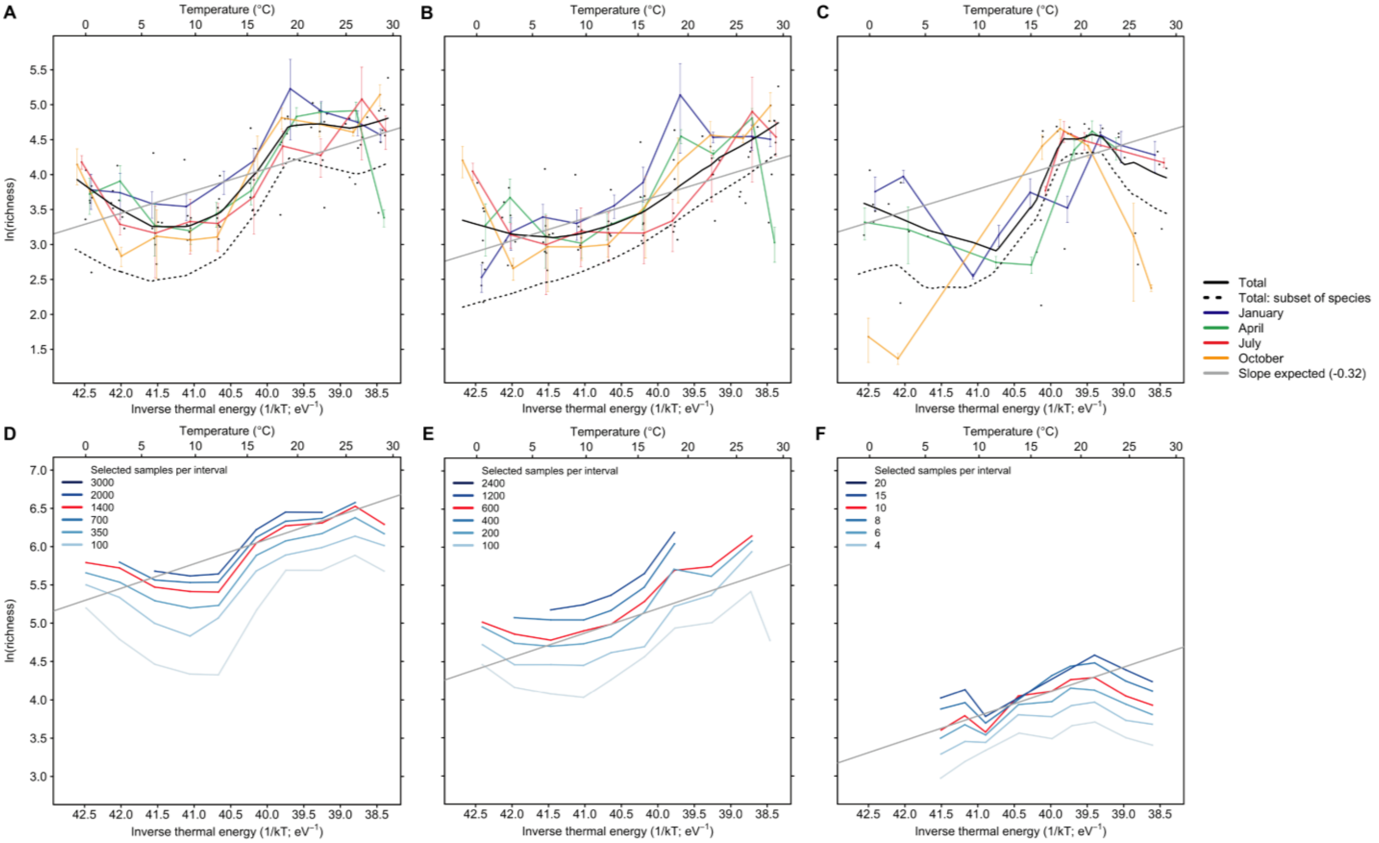


**Fig. S5. Species richness–temperature relationships derived from the observational raw data.** (**A** to **C**) Colored lines show the logarithmic species richness trends for individual months, using a resampling procedure with thermal bins chosen to be of equal size (~3.5° C; *n* = 10 bins). Phytoplankton species richness was obtained by summing up species from ten samples selected at random per thermal bin and month (*n* = 12 months). Dots, error bars, and lines are defined as for fig. S4. (A) Global results. (B) Northern hemisphere. (C) Southern hemisphere. The slopes estimated by linear regressions to the monthly richness data are –0.35, –0.36, and –0.29 in (A-C). Grey lines denote the slope predicted by metabolic theory (–0.32). (**D** to **E**) Colored lines highlight the richness emerging from annually pooled observational data, drawing different number of samples at random from within the thermal bins. (D) Global results. (E) Results for the Atlantic. (F) Results obtained from annually pooled, independent *in situ* data (Materials and Methods). Lines in (D to E) are not shown if the maximum sample numbers available per bin were insufficient.

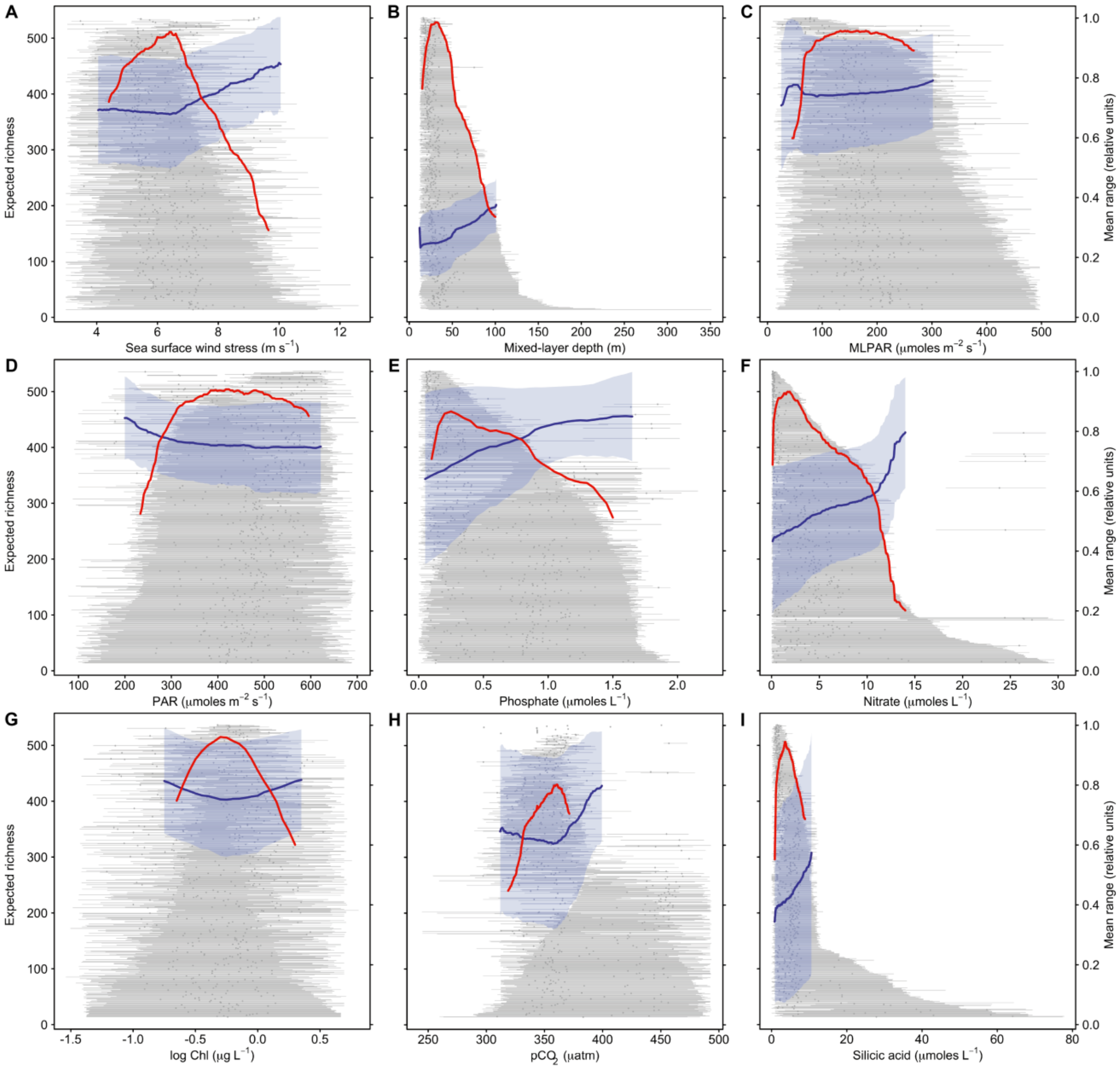


**Fig. S6. Species ranges for key environmental factors.** (**A** to **I**) Species' observed ranges are displayed by horizontal grey bars (minimum to maximum, dots for median). Vertical sorting is by descending range-size from bottom to top. Red lines indicate the expected richness defined as the number of species overlapping for each environmental condition. Blue lines indicate the average range width of the overlapping species (± 1 s.d., blue shading). Lines are shown for the parts of the gradients with higher confidence (Materials and Methods). Variables in **A** to **I** are the most powerful correlates of global SDM-based species richness besides temperature and latitude: MLPAR, mixed-layer photosynthetically available radiation; PAR, photosynthetically available radiation; Chl, chlorophyll a concentration; $pCO_2$, sea surface partial pressure of $CO_2$. Each panel displays the ranges for the 536 species that were also included in SDM analyses (GAM).

## Table S1. Fraction of equatorial species recorded at higher latitudes.

| Interval | Number of species recorded | %recorded at low latitudes (20°S - 20°N) |
|---|---|---|
| 80°N - 90°N | 155 | 56.1 |
| 70°N - 80°N | 241 | 63.5 |
| 60°N - 70°N | 303 | 79.5 |
| 50°N - 60°N | 383 | 80.4 |
| 40°N - 50°N | 545 | 77.4 |
| 30°N - 40°N | 754 | 69.4 |
| 20°N - 30°N | 578 | 89.1 |
| **20°S - 20°N** | **807** | **100.0** |
| 20°S - 30°S | 542 | 86.3 |
| 30°S - 40°S | 481 | 82.1 |
| 40°S - 50°S | 260 | 80.4 |
| 50°S - 60°S | 244 | 63.5 |
| 60°S - 70°S | 174 | 60.9 |
| 70°S - 80°S | 31 | 67.7 |
| 80°S - 90°S | – | – |

Absolute numbers and proportions of equatorial (low latitude) species (occurring between 20°S to 20°N) recorded within more poleward latitude bands (width of 10° latitude) in the observational data. The tropical reference band is highlighted in grey shade.

## Table S2. Single variable model skill for predicting species distributions and global richness.

| | Explanatory skill of the single variable in species models (mean ± s.d. across species)† | | | Explanatory skill of the single variable in global richness models | |
|---|---|---|---|---|---|
| Variable | GLM, adj.$D^2$ | GAM, adj.$D^2$ | RF, 1–OOB | GLM, adj.$D^2$ sample based†† (subset of species) | GLM, adj.$D^2$ SDM based |
| **T** | **0.30 ± 0.19** | **0.32 ± 0.18** | **0.72 ± 0.12** | **0.047 (0.108)** | **0.813** |
| **Wind** | **0.20 ± 0.16** | **0.21 ± 0.15** | 0.67 ± 0.10 | **0.042 (0.073)** | **0.621** |
| **$pCO_2$** | **0.19 ± 0.22** | **0.21 ± 0.21** | **0.70 ± 0.13** | 0.007 (0.004) | 0.192 |
| **$PO_4^{3-}$** | **0.15 ± 0.16** | **0.19 ± 0.15** | 0.67 ± 0.10 | 0.015 (0.024) | 0.427 |
| **N-star** | **0.15 ± 0.17** | 0.17 ± 0.17 | **0.70 ± 0.11** | 0.009 (0.019) | 0.037 |
| **PAR** | **0.15 ± 0.10** | 0.15 ± 0.10 | 0.65 ± 0.09 | 0.011 (0.019) | **0.591** |
| **S** | 0.14 ± 0.15 | **0.20 ± 0.17** | **0.69 ± 0.10** | 0.001 (0.002) | 0.086 |
| **Log $PO_4^{3-}$** | 0.14 ± 0.15 | 0.18 ± 0.16 | 0.67 ± 0.10 | 0.014 (0.016) | 0.366 |
| **$\Delta$MLD/$\Delta$t** | 0.13 ± 0.10 | 0.14 ± 0.10 | **0.70 ± 0.06** | 0.005 (0.008) | 0.075 |
| **Si-star** | 0.12 ± 0.11 | 0.14 ± 0.11 | 0.65 ± 0.09 | **0.027 (0.050)** | 0.201 |
| **$NO_3^-$** | 0.12 ± 0.15 | 0.13 ± 0.15 | 0.64 ± 0.10 | **0.016 (0.028)** | **0.473** |
| **Log Chl** | 0.11 ± 0.10 | 0.12 ± 0.10 | 0.65 ± 0.09 | **0.020 (0.053)** | 0.254 |
| **$Si(OH)_4$** | 0.11 ± 0.12 | 0.12 ± 0.13 | 0.65 ± 0.09 | 0.002 (0.011) | 0.120 |
| **MLPAR** | 0.10 ± 0.08 | 0.11 ± 0.09 | 0.63 ± 0.08 | 0.015 (**0.028**) | **0.699** |
| **Log $NO_3^-$** | 0.10 ± 0.14 | 0.13 ± 0.16 | 0.65 ± 0.10 | 0.009 (0.020) | 0.407 |
| **Log Si** | 0.09 ± 0.12 | 0.12 ± 0.13 | 0.65 ± 0.09 | 0.001 (0.009) | 0.086 |
| **Chl** | 0.09 ± 0.09 | 0.11 ± 0.10 | 0.65 ± 0.09 | <0.001 (0.010) | 0.122 |
| **Log MLD** | 0.09 ± 0.08 | 0.10 ± 0.08 | 0.68 ± 0.06 | 0.011 (0.006) | 0.359 |
| **MLD** | 0.09 ± 0.07 | 0.09 ± 0.07 | 0.68 ± 0.06 | 0.011 (0.005) | 0.369 |
| **$\Delta NO_3^-/\Delta$t** | 0.07 ± 0.10 | 0.09 ± 0.10 | 0.63 ± 0.09 | 0.002 (0.006) | 0.045 |
| **$\Delta$T/$\Delta$t** | 0.05 ± 0.07 | 0.06 ± 0.08 | 0.63 ± 0.09 | 0.001 (0.002) | 0.040 |
| **$\Delta PO_4^{3-}/\Delta$t** | 0.03 ± 0.06 | 0.05 ± 0.07 | 0.61 ± 0.07 | <0.001 (<0.001) | 0.054 |

Summary of the explanatory skill of single variables for the distribution of individual species in the observational data (performed independently of SDMs) and for the distribution of species richness. Species richness is both explained at the level of samples in the observational raw data ('sample based'; $n$ = 1300 species) and at the level of SDM results ('SDM based'; $n$ = 567 species). The five highest values (top five performing variables) per column are highlighted in bold font. For sample based richness, parentheses additionally show the results of individual variables for explaining the richness of the subset of species that were finally included in SDM richness analyses ($n$ = 536 species; GAM). GLM, generalized linear models. GAM, generalized additive models. RF, random forest models. Adj, adjusted. GLMs were built using linear and quadratic terms, unlike GAM and RF. Predictive skill was measured by the adjusted $D^2$ for GLM and GAM, and by the out-of-bag (OOB) error rate for RF. For units and full names of predictors, see Materials and Methods.

† Phytoplankton species ($n$ = 567) with at least 24 presences were considered for single variable species models. Background data for these models were sampled using group-specific target groups (Materials and Methods).

†† Species richness of individual samples was defined by species co-detected in space and time.

## Table S3. Contribution of sources to the phytoplankton dataset.

| Source | Number of observations (%unique to source) | Number of species (%unique to source) |
|---|---|---|
| GBIF* | 633,759 (60.8) | 1048 (28.1) |
| OBIS** | 640,271 (60.7) | 1140 (35.0) |
| MAREDAT# | 34,413 (88.2) | 115 (3.5) |
| Villar *et al.*☆ | 187 (100.0) | 82 (0.0) |
| **Total** | **1,056,363** | **1300** |

Number of observations (with % of observations unique to the source in parentheses) and the number of species (with % of species unique to the source in parentheses) retrieved from the four data sources used in this study.

* Global Biodiversity Information Facility (https://www.gbif.org, retrieved on 7 December 2015)

** Ocean Biogeographic Information System (https://www.obis.org, retrieved on 5 December 2015)

# MAREDAT (*37*)

☆ Villar *et al.* (*36*) using the records on *Bacillariophyceae* (Table W8 of ref. *36*) and *Dinoflagellata* (Table W9 of ref. *36*).

## Table S4. Statistics on data collected and species modeled within major taxon groups.

| Taxon | Source | Number of presence observations | Number of species in the data (modeled) | % total known (modeled‡) species number | Range (mean) of known marine species number | Share among modeled species |
|---|---|---|---|---|---|---|
| *Bacillariophyceae* | GBIF*, OBIS**, MAREDAT#, Villar *et al.*☆ | 546,058 | 482 (232) | 10 - 27 (6.8) | 1800† - 5000§ (3400) | 43.28 % |
| *Dinoflagellata* | GBIF*, OBIS**, Villar *et al.*☆ | 459,489 | 643 (258) | 36 (14.4) | 1780† - 1800§ (1790) | 48.13 % |
| *Haptophyta* | GBIF*, OBIS**, MAREDAT# | 27,342 | 127 (32) | 26 - 42 (8.9) | 300†,ǁ - 480§ (360) | 5.97 % |
| *Chlorophyta* | GBIF*, OBIS** | 501 | 25 (4) | 20 - 25 (3.5) | 100§ - 128† (114) | 0.75 % |
| *Chrysophyceae* | GBIF*, OBIS** | 1500 | 11(3) | 1 - 8 (0.6) | 130† - 800§ (465) | 0.56 % |
| *Cryptophyta* | GBIF*, OBIS** | 1311 | 4 (2) | 4 - 5 (2.2) | 78† - 100§ (89) | 0.37 % |
| *Cyanobacteria* | GBIF*, OBIS**, MAREDAT# | 19,574 | 4 (4) | 3 (2.7) | 150§ | 0.75 % |
| *Euglenoidea* | GBIF*, OBIS** | 583 | 2 (1) | 6 (3) | 30§ - 36† (33) | 0.19 % |
| *Raphidophyceae* | GBIF*, OBIS** | 5 | 2 (0) | 20 - 50 (0) | 4† - 10§ (7) | 0.00 % |
| **Total** | | **1,056,363** | **1300 (536)** | **8 - 12 (3.8)** | 11,200†,¶ - 16,940§ (14,145) | 100.0 % |

The table summarizes the observation numbers derived from the data sources with regard to nine major taxon groups and describes to what degree the observed and the modeled species in this study represent the total number of known species within the groups (for which exact species numbers are still debated; we therefore provide upper and lower bounds, and the mean value in parentheses). Further, the relative contribution of species modeled within each taxon group to the total number of species modeled is indicated. The estimates on the total known phytoplankton species include both coastal and open ocean taxa, whereas this study focuses on the open ocean only.

* Global Biodiversity Information Facility (https://www.gbif.org, retrieved on 7 December 2015)

** Ocean Biogeographic Information System (https://www.obis.org, retrieved on 5 December 2015)

# MAREDAT (*37*)

☆ Villar *et al.* (*36*)

† de Vargas *et al.* (*41*)

§ Falkowski *et al.* (*64*)

ǁ Jordan *et al.* (*65*)

‡ Parentheses indicate the number of species modeled (GAM) divided by the number of species known (mean) per taxon.

¶ This estimate excluded prokaryotes (*41*). A number of 150 prokaryotes (*64*) was added to this estimate to obtain the mean.
